# Performance of the Self‐Controlled Case Series With Active Comparators for Drug Safety Signal Detection Using Merative MarketScan Research Databases

**DOI:** 10.1002/pds.70250

**Published:** 2025-11-11

**Authors:** Astrid Coste, Angel Y. S. Wong, Francois Haguinet, Andrew Bate, Ian J. Douglas

**Affiliations:** ^1^ Department of Non‐Communicable Diseases Epidemiology LSHTM London UK; ^2^ GSK London UK

**Keywords:** claims data, pharmacoepidemiology, pharmacovigilance, real‐world data, self‐controlled case series, signal detection

## Abstract

**Background:**

Despite testing of epidemiological methods in US Claims databases for signal detection, such data sources have not become a routine capability. The Self Controlled Case Series (SCCS) is one of the most promising methods for drug safety signal detection using Real World Data, and incorporating active comparators could potentially improve its performance by addressing confounding by indication.

**Objectives:**

This study aims to evaluate the performance of the SCCS with and without active comparators for signal detection using US Merative MarketScan Commercial Claims and Medicare databases.

**Methods:**

We applied the SCCS to macrolide and fluoroquinolone antibiotics, using amoxicillin and cefalexin as active comparators. In total, 7 drugs and 30 outcomes from all organ classes were selected. We developed a reference set of 104 positive controls and 58 negative controls, using a taxonomy framework to ensure the selected drug outcome pairs are theoretically well suited to the SCCS design. A two‐year observation period with a 30‐day risk window after each dispensing was used. Diagnostic performance was measured using sensitivity and specificity with respect to the product labels.

**Results:**

The SCCS without active comparators achieved sensitivities of 0.73 and 0.72 and specificities of 0.68 and 0.62 in commercial and Medicare claims, respectively, for pairs with sufficient power. Active comparators increased specificity up to 0.84 and 0.86, respectively, in Commercial Claims and Medicare but decreased sensitivity to 0.45 and 0.36.

**Conclusions:**

MarketScan databases are potentially suitable for drug safety signal detection due to their large size and information contained. Using a carefully designed reference set of drug‐outcome pairs well suited to the study design, the SCCS, while imperfect, performed comparably to optimal settings identified in previously published studies. Active comparators did not enhance overall performance but showed improved specificity by better controlling confounding by indication at the cost of reduced sensitivity.

**Summary**

- The Self‐Controlled Case Series (SCCS) is a promising method for drug safety signal detection using Real‐World Data but has not been implemented routinely because of heterogeneous performance reporting.
- We explored SCCS performance in US claims data with and without active comparators using a tailored reference set based on macrolides and fluoroquinolones drug labels.
- The SCCS without active comparators achieved sensitivities of 0.73 and 0.72 and specificities of 0.68 and 0.62 in MarketScan Commercial Claims and Medicare, respectively, for pairs with sufficient power.
- Using reference set of drug‐outcome pairs well suited to the study design, the SCCS performed comparably to optimal settings identified in previous published studies. Active comparators did not enhance overall performance, but showed improved specificity by better controlling confounding by indication.

## 1 Introduction

US health insurance claims databases are widely used for the post‐marketing surveillance of drugs, especially with the development of the Sentinel initiative. Whilst Real World Data (RWD) sources have traditionally been used to test hypotheses arising from spontaneous reports (SRs), they can also play a complementary role in signal detection. US claims data have been used in 43 published signal detection studies in the past 20 years [1], including insurance company claims (e.g., Optum), large claims databases from employers, health plans and Medicare and Medicaid programs (Merative MarketScan), as well as claims and Electronic Health Records (EHRs) data sources from states and hospitals (e.g., Veterans Affairs Medical Center in Detroit, Michigan) [2, 3, 4]. However, signal detection using RWD remains at an investigation stage and has not become a routine capability because of insufficiently compelling method performance and lack of consensus on the best approach [5].

To determine the value of RWD for signal detection, there is a need to evaluate the performance of traditional epidemiological methods at finding alerts in a hypothesis‐free context. In practice, this is limited by the requirements of study designs, which can better accommodate certain types of drugs and outcomes. Previous RWD signal detection initiatives exploring method performance did not consider that epidemiological methods are differentially valid depending on the nature of the drug and outcome [6].

Among methods that have been evaluated for signal detection in RWD, the Self‐Controlled Case Series (SCCS) was one of the highest performing methods, with area under the receiver‐operator characteristics curves (AUCs) ranging from 0.57 to 0.74 in various studies, databases and settings [4, 7, 8, 9, 10]. Specifically in US Claims, the average AUC of SCCS across four outcomes and several drugs was 0.72–0.74 in the MarketScan Commercial Claims database and 0.70–0.72 in Medicare for optimal analytical settings [4, 11]. In both studies, only pairs with sufficient power were considered.

One of the advantages of self‐controlled designs is that time‐independent confounding is inherently accounted for because within‐person comparisons are made. However, they are still subject to time‐varying confounding, particularly by indication [12]. Active comparators can account for time‐varying confounding by incorporating comparator drugs with a similar indication as the drug of interest, which is not expected to cause the outcome under study. They have been successfully implemented with self‐controlled designs in hypothesis testing studies using Danish registry data [12], as well as in Hong Kong and British EHRs databases [13, 14], but not in US Claims databases. Active comparators with SCCS have not been evaluated for signal detection, so their impact on method's performance remains unknown.

We therefore aim to evaluate the performance of the SCCS with and without active comparators for signal detection in Merative MarketScan Commercial Claims and Medicare databases, with a reference set of drug‐outcome pairs theoretically well suited to the study design. Using a drug‐based approach, antibiotics were chosen for this study as they are prescribed in short courses, making them well suited to the SCCS design. Antibiotics have a well‐established safety profile since they have been on the American market for several decades, which is important for performance assessment.

## 2 Methods

### 2.1 Data Source

The MarketScan Research Databases contain individual‐level, de‐identified, healthcare claims information from employers, health plans, hospitals, and Medicare and Medicaid programs. The Commercial Claims and Encounters (CCAE) database contains data from active employees and dependents, and early retirees (non‐Medicare) for 38.7 million lives in the period 2019–2021. The Medicare database consists of data from active employees and retirees aged 65+, as well as dependents for 2.3 million in 2019–2021. Both databases include data from in‐ and out‐patient visits and prescription claims, as well as demographics, diagnoses, procedures, pharmacy claims and cost information [15]. Prescriptions are captured with the National Drug Code (NDC) classification and diagnoses and procedures with ICD10CM and ICD9‐CM. In this study, the Medicaid database was not used due to systematic differences in demographics, billing, and claims adjudication practices compared with the other two databases. We only used de‐identified patient‐level data; therefore, individual informed consent was not required.

MarketScan databases are large RWD sources. They benefit from employer‐provided data to minimise enrolment discontinuation due to changes in health plans. Some limitations of these data include a relatively short follow‐up time, as well as non‐nationally representative populations, because all patients are enrolled in employer‐based insurance. Only care consumptions leading to a claim are captured. Other medications, conditions or procedures may not be captured, such as over‐the‐counter medications.

MarketScan databases were used for performance assessment of methods for signal detection in the Observational Medical Outcomes Partnership (OMOP) initiative [4, 9], and are also used for routine drug surveillance as part of the Sentinel initiative, including signal detection experimentation [16].

### 2.2 Study Design

The SCCS is a case‐only design comparing the event rate during exposed and unexposed time within the same individual [11]. Since all comparisons are made within person, time invariant, between person confounding is inherently addressed. However, it remains vulnerable to time‐varying confounding. Active comparators have been recently implemented with SCCS [12] for epidemiological studies to reduce time‐varying confounding by indication. They could potentially improve the signal detection performance of SCCS further and, as such, have been implemented in this study. SCCS also relies on a range of assumptions, detailed in the Supporting Information.

### 2.3 Study Population and Design Choices

Details on study design choices can be found in the Supporting Information, as this study builds on a common protocol applied in three different data sources: the Clinical Practice Research Datalink (CPRD) and the French ‘Systeme National des Donnees de Sante’ (SNDS). Some adjustments were needed to analyse MarketScan data, in particular using different codelists.

The observation period lasted 2 years from 1 January 2017 to 31 December 2018. The risk window lasted 30 days and began on the day after the prescription was filled. We conducted a crude primary analysis assuming there were no time‐varying confounders. The population consisted of all adults with at least one oral macrolide (azithromycin, erythromycin, clarithromycin) or fluoroquinolone (FQ) (ciprofloxacin, ofloxacin, levofloxacin, moxifloxacin) prescription during the observation period. Participants were eligible for inclusion at the latest of 1 year post‐registration or their 18th birthday. Two active comparators (amoxicillin and cefalexin) were chosen from different antibiotic classes but with similar indications to macrolides and fluoroquinolones.

### 2.4 Reference Set

A reference set was designed for this study using a taxonomy framework to ensure the selected drug outcome pairs are well suited to the SCCS design. The full process has been detailed in a previous paper [17]. The reference set contains 104 positive controls (drug‐outcome pairs) and 58 negative controls in total, based on 30 outcomes from all organ classes.

Positive and negative control outcomes were selected among outcomes present on UK labels, on an individual drug basis. Only clinically important outcomes meeting the SCCS requirements and a priori appropriately captured in MarketScan were considered in the reference set, based on a classification included in the taxonomy framework. UK labels presented only minor differences with the US versions and were used to harmonise the reference set across the three studies.

The choice of negative controls is particularly challenging given the evolving nature of drug safety knowledge, both in terms of what causes or does not cause health outcomes. In this study, negative control outcomes were selected using the same list of positive control outcomes since they are theoretically well suited to the SCCS design. We chose negative control outcomes specifically for each drug, requiring that the outcome is not labelled as an expected Adverse Drug Reaction (ADR) for any of the drugs in the same drug class. For example, amnesia is a positive control (listed ADR) for moxifloxacin but is not on any of the macrolides' labels and, as such, is a negative control for all macrolides. Two negative controls were added on top of this list, which have been considered in previous signal detection studies: hip fracture and upper gastrointestinal bleeding [9, 18].

### 2.5 Statistical Analyses and Measures of Performance

We used conditional Poisson regression to estimate incidence rate ratios and incorporated active comparators using both the simple ratio and effect modifier models. Performance was measured using sensitivity and specificity with respect to the reference set and computing the resulting area under the curve (AUC) of the receiver operating characteristics (ROC).

A common reason, even in a large US Claims database, for the inability to highlight safety signals is insufficient statistical power for rare outcomes. We therefore looked primarily at the relative performance of the drug‐outcome pairs where there was apparently sufficient power for SCCS with and without the application of an active comparator. We considered satisfactory power for drug‐outcome pairs with at least five events in the risk window, and where the upper bound of the 95% confidence interval Risk Ratio (RR) was < 2; effects with no evidence, e.g., RR = 1.55 (95% CI: 0.90–2.10), would not be considered satisfactory power. This also enabled comparison of SCCS performance between databases of different countries, the results of which are described in another publication.

### 2.6 Length of the Risk Window

Reported times to ADR onset following antibiotic initiation vary by organ system, with median times to development of 5–15 days across all ADRs in US EHRs [19]. Specifically for fluoroquinolones, a majority of ADRs started in the first 7 days after starting the drug [20]. Therefore, we explored the impact on SCCS performance of a shorter 15‐day risk window in a secondary analysis, which could reduce the dilution of effect of a longer risk window.

## 3 Results

1 915 466 patients met the inclusion criteria in the Commercial Claims database and 312 783 in Medicare. Mean age at cohort entry was 45.7 in CCAE and 76.3 in Medicare. There were more women than men in the population, with a 61.0/39.0 ratio in CCAE and 56.4/43.6 in Medicare. Average length of follow‐up was 628.4 days in CCAE and 600.3 days in Medicare. Medicare patients had more antibiotic prescriptions on average in the observation period than CCAE patients, with 3.1 and 2.7 prescriptions, respectively. The characteristics of the population by drug class in CCAE and Medicare can be found in Tables 1 and 2.

**TABLE 1:** Key characteristics of the CCAE population by drug class.

	FQs	Macrolides	Amoxicillin	Cefalexin
Population	384 258	716 350	1 116 037	436 772
Age at cohort entry				
Mean (SD)	48.9 (11.9)	45.6 (13.0)	45.4 (13.2)	45.7 (13.4)
Median (IQR)	52 (17)	47 (22)	48 (21)	48 (21)
Sex (%)				
Female	63.8	64.2	61.3	60.8
Male	36.2	35.8	38.7	39.2
Mean length of Follow‐Up (days)	635.3	641.0	635.9	632.8
Number of antibiotic prescriptions^*^				
Mean (SD)	3.1 (2.4)	2.6 (2.0)	2.5 (1.9)	2.7 (2.1)
Median (IQR)	2 (3)	2 (2)	2 (2)	2 (2)

* Of all antibiotics considered in this study.

**TABLE 2:** Key characteristics of the medicare population by drug class.

	FQs	Macrolides	Amoxicillin	Cefalexin
Population	107 920	95 159	160 481	92 119
Age at cohort entry				
Mean (SD)	77.3 (8.3)	75.5 (7.7)	75.6 (7.7)	77.4 (8.3)
Median (IQR)	76 (13)	74 (12)	74 (12)	76 (12)
Sex (%)				
Female	58.9	40.6	54.2	56.5
Male	41.1	59.4	45.8	43.5
Mean length of Follow‐Up (days)	597.1	624.0	616.0	606.4
Number of antibiotic prescriptions^*^				
Mean (SD)	3.2 (2.6)	3.0 (2.5)	3.0 (2.5)	3.1 (2.7)
Median (IQR)	2 (3)	2 (3)	2 (3)	2 (3)

* Of all antibiotics considered in this study.

### 3.1 Simple Ratio Model

The overall sensitivity and specificity of the SCCS without active comparator were 0.66 and 0.74, respectively, in commercial claims (Table 3), and 0.53 and 0.77 in Medicare (Table 4), for all drug‐outcome pairs with at least one event during the risk period. These measures increased when restricted to drug‐outcome pairs with satisfactory power: overall sensitivity and specificity of 0.73 and 0.68 in commercial claims and 0.72 and 0.62 in Medicare. Specificity increased up to 0.84 in commercial claims with amoxicillin as an active comparator; however, sensitivity decreased to 0.45. Both databases achieved the same result for 71 of the 84 drug‐outcome pairs (84.5%) with sufficient power.

**TABLE 3:** Measures of performance for the SCCS in MarketScan commercial claims—simple ratio model.

	All pairs with at least one event during the risk or baseline period^a^			Pairs with sufficient power		
	No comparator	Amoxicillin	Cefalexin	No comparator	Amoxicillin	Cefalexin
Number of pairs	137	137	137	116	112	106
Sensitivity	0.66	0.38	0.36	0.73	0.45	0.43
Specificity	0.74	0.85	0.87	0.68	0.84	0.83
PPV	0.81	0.83	0.83	0.83	0.85	0.83
NPV	0.55	0.44	0.43	0.55	0.44	0.43
AUC	0.70	0.62	0.62	0.71	0.65	0.63

^a^ 25 drug‐outcome pairs not looked at because of no events.

**TABLE 4:** Measures of performance for the SCCS in MarketScan medicare—simple ratio model.

	All pairs with at least one event during the risk or baseline period^a^			Pairs with sufficient power		
	No comparator	Amoxicillin	Cefalexin	No comparator	Amoxicillin	Cefalexin
Number of pairs	128	128	128	84	84	84
Sensitivity	0.53	0.31	0.26	0.72	0.44	0.36
Specificity	0.77	0.87	0.91	0.62	0.83	0.86
PPV	0.80	0.81	0.84	0.81	0.83	0.84
NPV	0.49	0.42	0.41	0.50	0.43	0.39
AUC	0.65	0.59	0.59	0.67	0.64	0.61

^a^ 34 drug‐outcome pairs not looked at because of no events.

### 3.2 Nested Model

There were very minor differences between the simple ratio model and the nested model. All results of the nested model are included in the Supporting Information.

### 3.3 Sensitivity Analyses: 15 Days Risk Window in Commercial Claims

Compared with the primary analysis which used a 30‐day risk window, the sensitivity slightly increased (0.79) using a 15‐day risk window for pairs with sufficient power, but the specificity decreased to 0.61 (Table 5). For example, the RR obtained for the pair moxifloxacin‐cellulitis (negative control) was 1.07 (95% CI: 0.78–1.46) in the 30‐day risk window analysis; this increased to 1.46 (95% CI: 1.02–2.08) in the 15‐day risk window. Using active comparators, the sensitivity and specificity were similar to the primary analysis.

**TABLE 5:** Measures of performance for the 15 days risk window analysis in MarketScan commercial claims.

	All pairs with at least one event during the risk or baseline period			Pairs with enough power		
	No comparator	Amoxicillin	Cefalexin	No comparator	Amoxicillin	Cefalexin
Number of pairs	136	136	136	107	105	105
Sensitivity	0.65	0.37	0.36	0.79	0.46	0.45
Specificity	0.68	0.82	0.86	0.61	0.80	0.87
PPV	0.78	0.78	0.82	0.80	0.82	0.86
NPV	0.53	0.43	0.44	0.60	0.42	0.46
AUC	0.67	0.60	0.61	0.70	0.63	0.66

### 3.4 Contrast Between Analytical Approaches With and Without Active Comparators

**FIGURE 1:**
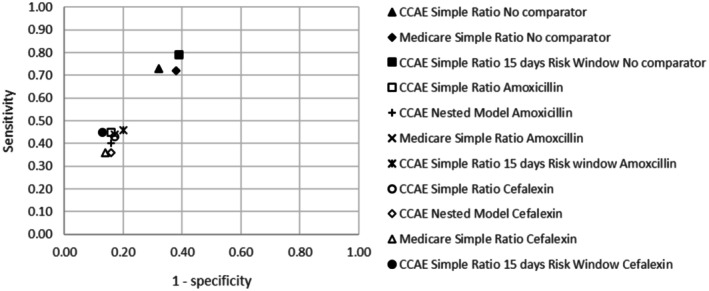
Performance plot for different settings. CCAE, commercial claims and encounters.

Figure 1 shows two clusters of performance when plotting the performance of different analytical approaches and databases in this study. When no active comparator was used, the sensitivity was highest, but the specificity decreased compared to analyses using an active comparator.

## 4 Discussion

The performance of SCCS for signal detection in CCAE, when using a carefully designed reference set of drug‐outcome pairs well suited to the study design for pairs with enough power, showed a 0.73 sensitivity and a 0.68 specificity in the absence of an active comparator. It was slightly lower in Medicare (0.72 sensitivity and 0.62 specificity), which is a smaller database. Whilst specificity increased slightly when active comparators were applied, sensitivity was much reduced.

Power was satisfactory for 71.6% of all drug‐outcome pairs included in the reference set in Commercial claims, and for 51.9% in Medicare. Azithromycin was the most commonly used drug of interest, which also corresponded to the highest performance of the method (0.77 sensitivity and 0.79 specificity in CCAE, 0.77 and 0.92, respectively in Medicare). We were not able to perform the analysis for ofloxacin because of the lack of power. This is because ofloxacin is mostly prescribed as eye and ear drops in the US [21], formulations excluded from this study.

The results in CCAE and Medicare correlated in 84.5% of cases, providing reassurance on the performance of SCCS as the two populations differ substantially. The main differences were observed with outcomes occurring more frequently in the older population covered in Medicare. For example, despite Medicare being much smaller in size than CCAE, some outcomes had greater sample sizes in Medicare than CCAE (e.g., hip fracture).

MarketScan databases were previously included in signal detection performance assessment initiatives. Two studies evaluated performance on 4 and 9 outcomes, respectively, and a range of drugs [9, 11]. Reported AUCs for optimal design choices ranged between 0.72 and 0.74 in CCAE and 0.70 and 0.73 in Medicare, although this varied significantly by outcome. Lowest reported AUCs for each outcome and database were lower than 0.3, and many combinations of settings led to AUCs < 0.5. Our study applied a single analytical setting as this is practical for signal detection and found slightly lower AUCs than the best performing settings in the studies above, with 0.71 in CCAE and 0.67 in Medicare, but significantly higher AUCs than all other settings. We used a different reference set to the OMOP project and therefore potentially different imbalances between positive and negative controls in terms of raw counts and relative sample sizes.

### 4.1 Reasons for Missed Positive Controls

Since the sensitivity of the method is one of the most important measures of performance for signal detection, we looked for reasons for missed positive controls. Notably, the reference set was solely derived based on drug labels. While labels provide easily accessible and harmonised information on ADRs, they also include variable levels of evidence of a causal association, as well as broad terms leading to unspecific codelists. As an example, fluoroquinolone labels include ‘tendinitis’ as an ADR, but published studies stated that a vast majority of cases occur in the Achilles tendon [22]. This lack of granularity can be an issue in signal detection as the effect of some true causal associations would be diluted. We note there is a varying level of predictive value of code lists in US insurance claims reflecting underlying disease [23], and while the validation of ICD codes for signal detection is limited, clearly inaccurate coding would impact signal detection capability.

### 4.2 Use of Active Comparators

Active comparators enabled an increase in specificity, which can be explained by a reduction in confounding by indication, particularly for certain outcomes (e.g., pneumonia and cellulitis). However, the sensitivity of the method was greatly decreased due to the high proportion of outcomes with strong evidence of elevated RRs when using active comparators (73% for both amoxicillin and cefalexin in CCAE). Moreover, the exposed populations could potentially differ between the investigated products and the active comparators; e.g., some antibiotics may be prescribed as second‐line therapy to patients with different comorbidities compared to the comparator drugs. For these reasons, active comparators need to be carefully selected, ideally on an individual drug‐outcome pair basis, to be best suited to the indications of the drug of interest. However, this is impractical in signal detection when evaluating multiple medications, implying that active comparators may not perform well in a signal generation context. There are also practical challenges for signal detection, particularly with newer products where identifying an appropriate comparator may be especially challenging.

When there was no confounding by indication, defined as a null association between an outcome and an active comparator, and a sufficient sample size, using an active comparator had no impact on the sensitivity of SCCS. When power was relatively lower, but still sufficient as defined in this study, we observed a decrease in sensitivity when using an active comparator. For example, the sensitivity decreased by 25% for fluoroquinolones with cefalexin as the comparator, although the analysis was conducted on a small number of drug‐outcome pairs. The minimum power threshold applied in this study may partly hide the decrease in sensitivity due to using an active comparator, but allowed us to focus on product‐events where the ability of the active comparator to address the indication bias had a better chance to be highlighted.

### 4.3 Length of the Risk Window

Signal detection aims for a simple design to screen a large number of drugs and/or outcomes in a single study. Ideally, the same risk window should apply to all the outcomes, but this choice should not be made arbitrarily. A risk window that is significantly longer than the period of influence of the exposure on the risk of the outcome will cause a dilution of the effect, and only the strongest signals stand out. In contrast, if the risk window is too short, power could be decreased and the effect diluted. When reducing the risk window from 30 to 15 days, we observed an increase in sensitivity of 5.8% in CCAE without an active comparator, and a decrease in specificity of 7.3%. Some outcomes had a higher IRR in days 16–30 after dispensing compared to days 1–15, especially longer onset or dose‐dependent ADRs e.g., ciprofloxacin and tendon rupture [24, 25]. Therefore, careful consideration is needed when defining the risk window in a comprehensive signal detection context and speaks to the value of signal detection methods that consider varying risk windows in semi‐automated manners [26, 27].

### 4.4 Strengths and Limitations of This Study

This study demonstrated the feasibility of signal detection in MarketScan databases. Despite including some relatively rarely prescribed drugs and rare outcomes, power was still adequate for most of the reference set. Power was a limitation for some drugs (e.g., ofloxacin) and outcomes (e.g., pancytopenia), either because they were rare or undercaptured in MarketScan databases.

The reference set included 30 outcomes from all organ classes, which is larger than other signal detection performance assessment studies. Although we only considered antibiotics, our results should generalise to any drug well suited to the SCCS requirements. A novel consideration in signal detection is to select drugs and outcomes meeting the assumptions and requirements of the study design, which has not been done in previous studies. Similarly to good practice in a hypothesis testing context, such considerations should be applied to signal detection studies, both for performance assessment and real‐life studies, to avoid spurious or missed alerts from inappropriate outcomes.

Our reference set was based on outcomes that were already present on drug labels at the time of the observation period. We did not explore whether the method can highlight signals before they were added to the drug labels as information on addition dates was not publicly available. Moreover, ADRs were included in labels with various levels of evidence, and some may not represent true causal associations. We derived the reference set from UK labels, for coherence with other studies under the same project, although the differences with US labels are minor since antibiotics have well‐established safety profiles.

Ideally, only a part of the data should be used for signal detection and other parts could be withheld for further signal assessment studies, using data partitioning [27]. Since Medicare is a smaller database, its entire population might be needed to detect all potential signals in a study. There is then the possibility of using other US claims for evaluation studies, recognising some potential overlap in the populations.

Finally, for signal detection in RWD to become widespread, using the SCCS or other methods, more research is needed to better understand the relative strengths or weaknesses of RWD compared to SRs data for drug safety signal detection.

## 5 Conclusions

The performance of the SCCS for drug safety signal detection in MarketScan databases, when tailoring the choice of the drug outcome pairs to the chosen study design, is in line with the best performing findings from previously published studies. The application of active comparators partially improved the handling of confounding by indication but also reduced the proportion of positive controls correctly identified. As a result, they appear to be more useful in a hypothesis testing context rather than for signal detection using SCCS.

### 5.1 Plain Language Summary

We explored the performance of a self‐controlled method, where risks of outcomes (potential adverse events) are compared between different time points in each patient, for the detection of Adverse Drug Reactions using health claims databases. We also explored the added value of a recent methodological development, active comparators. These are drugs used to treat similar health conditions but not reported to have adverse events of interest and can help address possible bias. We looked at the ability of the method to identify known drug side effects for commonly prescribed antibiotics. The method was able to give a correct result for 73% of positive controls (known adverse drug events) and 68% of negative controls (drugs known not to be associated with certain outcomes) in the Commercial Claims database, when limited to drug‐outcome pairs with a satisfying number of occurrences. In the Medicare database, the proportion of positive controls correctly identified was 72%, and 62% for negative controls. Overall, the performance of the method is towards the higher end of estimates from previous studies. Active comparators reduced the number of positive controls identified but improved correct identification of negative controls.

## Ethics Statement

As we used de‐identified patient‐level data, individual informed consent was not required. The study was approved by the London School of Hygiene and Tropical Medicine Research Ethics Committee (Reference: 27650).

## Conflicts of Interest

A.C. is funded by a GSK PhD studentship to undertake this work. A.B. is an employee of GSK and holds stocks and stock options. F.H. is an employee of GSK and holds financial equities in GSK. I.J.D. holds grants and shares from GSK. GSK markets the following drugs: Augmentin. However, A.B. and F.H. did not actively participate in the assessment of the labels and choice of outcomes for this methodological study.

## Supporting information


**Data S1:** Supporting Information.
