# Supplementary material for: Performance of the Self‐Controlled Case Series With Active Comparators for Drug Safety Signal Detection Using Merative MarketScan Research Databases
Source: Pharmacoepidemiol Drug Saf. 2025 Nov 11;34(11):e70250. doi: 10.1002/pds.70250 (PMC12603968; doi:10.1002/pds.70250)
Supplement: Supplementary file 1 — Data S1: Supporting Information. [file PDS-34-e70250-s001.docx]

## Main assumptions of the SCCS method

- Conditionally independent events [2]. In the main analysis, we only considered the first occurrence of an event to meet this assumption [1].
- An event does not influence subsequent exposures. A 30-day pre-exposure window was introduced to acknowledge that this assumption may be temporarily violated [1].
- An event does not influence the end of the observation period [3]. We ruled out outcomes leading to modest to large increases in mortality to meet this assumption in this study.
- Since accurate timing of exposure and outcome is crucial to SCCS [2], this method is better suited to transient exposures and acute outcomes. In this study, we considered antibiotics, which are usually prescribed in courses of less than 10 days. All chosen outcomes are acute, and we anticipate minimal misclassification of the time of occurrence.

## Details on multiple prescriptions and overlaps

If there were two or more prescriptions of the same drug with a gap shorter than 30 days, they were considered as continuous exposure with an extension of the risk window ending 30 days after the start of the last exposure. If the gap was longer than 30 days, the exposures were treated separately (Figure 2). When there were two or more concomitant or overlapping exposures of different antibiotics of interest, the corresponding risk periods were excluded. A 30-day pre-exposure window was introduced before every exposure due to the assumption that the outcome does not affect future exposure [1]. In the case of an overlapping risk period and pre-exposure risk window, the overlapping period was considered as risk period and the pre-exposure period was shortened.


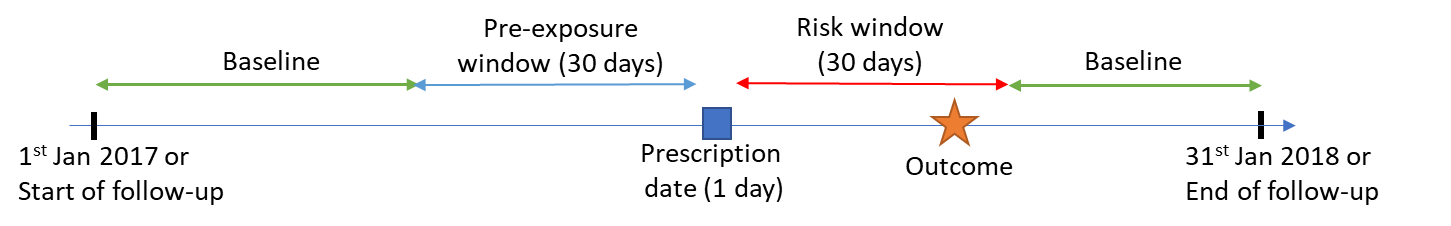


**Figure 1 – Summary of study design choices**


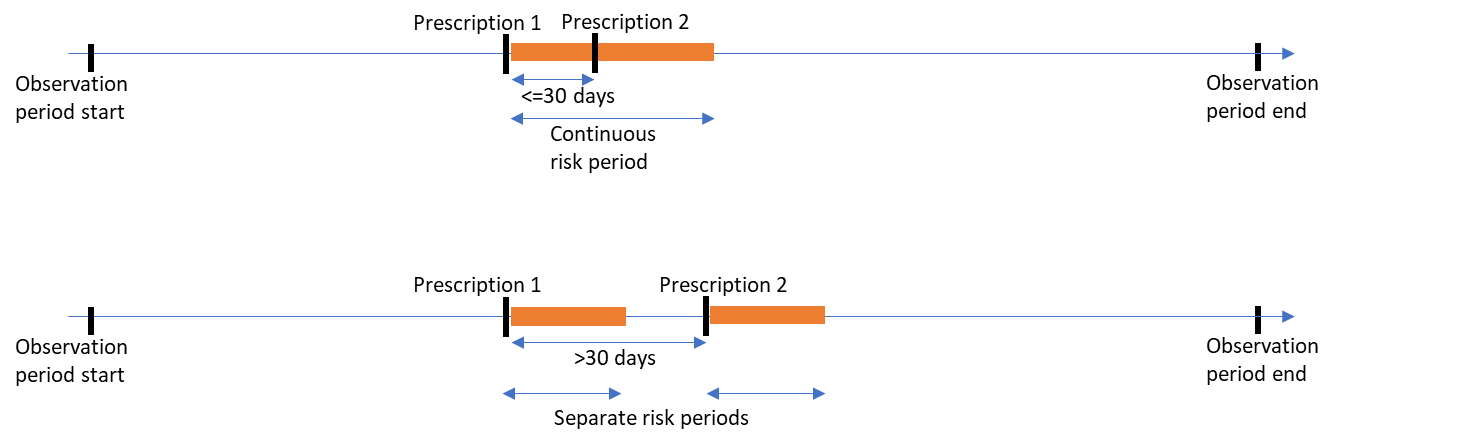
**Figure 2 – Summary of the risk periods for the study in the case of multiple prescriptions**

## Table - Measures of performance for the SCCS in MarketScan Commercial claims – Nested model

|  | All pairs with at least one event during the risk period | | Pairs with enough power | |
| --- | --- | --- | --- | --- |
|  | **Amoxicillin** | **Cefalexin** | **Amoxicillin** | **Cefalexin** |
| **Number of pairs** | 136 | 135 | 116 | 115 |
| **Sensitivity** | 0.35 | 0.32 | 0.40 | 0.36 |
| **Specificity** | 0.85 | 0.85 | 0.84 | 0.84 |
| **PPV** | 0.81 | 0.79 | 0.83 | 0.82 |
| **NPV** | 0.43 | 0.42 | 0.41 | 0.40 |
| **AUC** | 0.60 | 0.59 | 0.62 | 0.60 |

## References

1. Petersen I, Douglas I, Whitaker H. Self controlled case series methods: an alternative to standard epidemiological study designs. Br Med J [Internet]. 2016; Available from: http://dx.doi.org/10.1136/bmj.i4515

2. Zhou X, Douglas IanJ, Shen Rongjun, Bate Andrew, Douglas IanJ, Bate Andrew. Signal Detection for Recently Approved Products: Adapting and Evaluating Self-Controlled Case Series Method Using a US Claims and UK Electronic Medical Records Database. Drug Saf [Internet]. 2018 [cited 2021 Jul 13];41:523–36. Available from: http://rd.springer.com/journal/40264

3. Whitaker HJ, Ghebremichael-Weldeselassie Y, Douglas IJ, Smeeth L, Farrington CP. Investigating the assumptions of the self-controlled case series method. Stat Med. 2018;37:643–58.
